# Breathing atmospheres of failure: The matter of pollution in Beirut

**DOI:** 10.1177/23996544251394575

**Published:** 2025-11-06

**Authors:** Tiina Järvi, Mikko Joronen

**Affiliations:** 1 Geographies of Coloniality and Everyday Violence Research Group / Space and Political Agency Research Group, 7840 Tampere University, Tampere, Finland

**Keywords:** atmosphere, breathing, pollution, Beirut, failure, state neglect

## Abstract

This paper examines the atmospheric materialisation of failure in the context of Lebanon’s electricity infrastructure. Thinking through the rise in pollution levels in Beirut – which has been connected to the increased reliance to diesel-powered generators – the paper argues that the dysfunctional electricity sector and the polluting materiality it generates should be approached atmospherically. The paper shows, firstly, how the chronic state neglect of the electricity infrastructure – the entanglements of corruption, geopolitics, wasta networks, and multiple crises – remains ‘in the air’ as generators emit dangerous amounts of carcinogenic particles, enveloping Beirut into a toxic atmosphere. Secondly, the paper foregrounds the body’s immersion in its aerial environments through breathing. We argue that such immersion constitutes pollution as a form of embodied atmospheric failure, stemming from the state’s materialised (in)actions. By elaborating how this neglect permeates daily life – not only through affective entanglements, social inequalities and daily rhythms of power outages, but crucially through immersion in material atmospheres – the paper shows how air is embodied and existential, but also political, constituting a medium that allows pollution to encircle those dwelling in the city. Thirdly, the paper invites us to reconsider infrastructural failure and state neglect by examining how atmospheric pollution becomes an embodied part of city life. This, we argue, raises further questions about the allochronic temporalities of moving air and the challenging political terrain revealed by the aerialisation of infrastructural failure.

## Introduction

On 5 March 2024, a Lebanese civil society initiative *Live Love Beirut* published a series of photos on their Instagram page. The photos depict a view over Beirut, taken from a nearby town Broummana, located on a mountain slope east from the capital. The caption marvels at the view, with a quote from the photographer: ‘You can get incredible views just about anywhere in Lebanon, but I think this view sums up so much about the country’. The sentence was followed by a heart emoji and the flag of Lebanon, suggesting the sincerity of the statement. Yet, there was a dissonance between the photo and the caption: a thick layer of black smog hovered over the capital city, dimming the skyscraper silhouette the photos depicted. In addition to praise and admiration, many recognised this disconnect in the comments below the post. They shared their dismay over the dangerous amount of pollution in the air and made fun of the caption with typical Lebanese sarcasms: ‘Yes sums up the almost constant smog hovering over Beirut lol’, ‘When you pay your graphic designer in LBP .. lol’^1^ and ‘The air cover you actually have to chew before you breathe’ (Live Love Beirut, 2024).

Like many other cities (Elsom, 1996; Sicard et al., 2023), Beirut has long suffered from bad air quality and smog clouds (Chaaban, 2008). When living in Beirut in the early 2010s, Järvi remembers how disheartening it was to observe Beirut from afar. A black haze blurred the silhouette of the high-rise landscape, revealing the toxicity of the air one had to breathe in the city. Heavy and chaotic traffic with an aging vehicle fleet, emissions from industry as well as from waste burning have all contributed to the high pollution levels in the city (Azar and Azar, 2016; Baayoun et al., 2019; HRW, 2017). These levels are occasionally heightened by more violent sources, such as the polluting shockwave of the 2020 Beirut port explosion and the toxic remnants of Israel’s aerial strikes. However, a major and constant contributor has been the diesel-powered generators used to compensate for the failing electricity grid. Over the past 5 years, the air quality has plummeted to dangerous levels, largely due to increased reliance on the generators to provide electricity during the grid outages. Before the economic crisis that began in 2019, the generators usually ran 3 hours a day (in central Beirut), but since then the situation has deteriorated considerably. As Lebanese Lira lost its value, the state cut back from the subsidies that had kept the electricity sector afloat, turning diesel-run generators the main source of electricity from 2021 onwards. The same year, the usage typically ranged between 15 and 20 hours a day. A study conducted by American University of Beirut found that this reliance has had severe consequences on the air quality. Carcinogenic particles in the atmosphere have doubled since 2017. Cancer cases are on the rise as are respiratory diseases, with patient getting younger and the cases more aggressive (Mehdy, 2024). Consequently, in Beirut – as arguably in many other places – high pollution levels reflect the toxic atmospheres of the Anthropocene but are also deeply entwined with the specificities of the country’s political conditions.

In this article we interrogate air pollution as a localised material and atmospheric manifestation of state negligence. We argue that the focus on respiratory embodiment of atmospheric (or aerial) materialities can effectively help in understanding the state’s infrastructural negligence as an *atmospheric mode of failure.* This failure manifests through the contamination of those aerial conditions that shape the inhabitation of the breathing and sensing body. We build upon the ongoing geographical work on ‘material atmospheres’ (see Adey, 2015; Fregonese, 2017; McCormack, 2008; Nieuwenhuis and Nassar, 2018), rethinking particularly how pollution functions as atmospheric particles embodied in breathing (see Joronen, 2023; Nieuwenhuis, 2016; Stamatopolou-Robbins, 2022). We underline how the atmospheric materiality of pollution relates to the respiratory vulnerability of the body, the body embodying the ‘aerialisation’ of infrastructural failure, which results from state’s inability to provide stable and uninterrupted flow of electricity. We further touch upon how the material atmospheres become entangled with prevalent affective atmosphere shaped by the state’s inability to resolve the situation. There is failure *in the air* in Lebanon, not only as an affective reaction to the country's political realities (Musallam 2020)*,* but also, we argue, in a profoundly material manner.

In order to develop a grounded conceptualisation of pollution as an atmospheric failure, we draw on ethnographic fieldwork materials from everyday encounters and participatory research conducted in Beirut in 2024. In addition, we incorporate a range of supplementary sources, including more formal interviews conducted during the 2024 fieldwork, archival materials such as reports, news articles, and social media content, field diaries of Järvi’s earlier fieldwork in Lebanon (2015, 2019, 2023), and autoethnographic reflections on Järvi’s periods of living in the city (2013, 2014–2015). Rather than departing from the theorisations of air and pollution, we start from what the field incites us to think. The aim is to provide, through selected cases and ethnographic vignettes on informal encounters and observations, a space for conceptualising pollution as an embodied atmospheric failure. With this bottom-up approach our aim is hence not to provide a paper based on thick ethnographic account of pollution in Beirut, but rather to tease out and conceptualise the material, atmospheric and embodied aspects of state infrastructural failure materialising ‘in the air’. We start the paper with an overview of current and historical infrastructural conditions behind the increasing pollution levels in Lebanon and focus especially on how they relate to the increasing use of the diesel generators. In the second section, we use the fieldwork materials to elaborate the everyday atmospheres of dwelling with pollution in Beirut. Based on these elaborations, the third section moves to conceptualise *pollution as a mode of atmospheric failure*. Instead of using failure to describe the functioning of state governance (Leshem, 2024; Rose, 2014) or the infrastructural shortcomings (Alda-Vidal et al., 2023; Nassar, 2023), we argue that when thinking failure, the focus should be extended to atmospheric conditions. By looking at ways in which breathing bodies are negatively immersed in aerial materialities of state neglect or downright failure, we further conceptualise failure beyond the affirmative interpretations (Martínez, 2019; Mica et al., 2023; Osborne, 2019) and show how its allochronic and atmospheric formations make it a more challenging site for politics.

## The politics of pollution: Wasta, neglect and economics of collapse in Lebanon

Crumbling electricity infrastructure is a nuisance that has long framed the daily flows in Lebanon. Unreliable and inadequate, it constitutes a glaring example of the infrastructural decay but also of the political mismanagement and networks of wasta – meaning personal networks and connections that are used to gain favours – that lie behind it. While less life-threatening than other infrastructural issues (such as collapsing buildings, Farhan, 2024; Raydan, 2024), less in-your-face than the 2015 waste crisis that enveloped the capital with a foul smell when trash piled up in the streets, and less shocking and violent than the Beirut Port Explosion that momentarily put the city off-hinge in August 2020, the enduring reality of the failing electricity sector emerges from the same political climate of negligence and impunity. The problems of electricity distribution intertwine with the state provider Électricité du Liban (henceforth EDL), which together with its predecessors has been at the centre of controversies, both due to their inadequate operations and wider political struggles and calculations (Abu-Rish, 2014a; Verdeil, 2016). Dysfunctions and glitches have always been part of Lebanon’s electricity landscape, and the country has never enjoyed uninterrupted service nor stable voltage. Prescheduled power outages, for instance, have been the reality of electricity distribution since 1952 (Abu-Rish, 2014b). Consequently, the everyday has been constituted out of the mundaneness of outages. They structure people’s actions in myriad ways as quotidian tasks have been composed around their rhythms. Household chores have been organised according to the national electricity distribution, as the supplementary power sources do not usually provide high-enough voltages to run several bigger appliances simultaneously. The limited ambers mean that people have to prioritise their electricity usage or to postpone tasks until electricity from the state grid is available.

Escalations of violence – the civil war (1975–1990), the July War (2006) and Israel’s ongoing bombardment (2023–)^2^ being the most destructive ones – have caused significant damage to electricity infrastructure (Amnesty, 2006; Verdeil, 2016) and contributed to the continuous need for supplementing electricity providers (Abi Ghanem, 2018; Nucho, 2022). However, the reasons for the current decline in hours of EDL electricity lie not in war but in economic conditions. In 2021, the economic crisis that had started in latter half of 2019 had exacerbated so that the state was unable to purchase the needed fuel to keep the electricity production running. This resulted in large-scale blackouts and reduction in EDL electricity provision to a few hours a day. The difficulties in securing enough currency to keep the fuel-dependent system running have continued since, and in August 2024 EDL informed that it was, again, running out of fuel, which resulted in nationwide blackouts (HRW, 2024). These outages in EDL-provided electricity have been, and continue to be, a source of inequality. Those with financial means can afford alternative electricity sources and are therefore less affected by the disturbances caused by inadequate service. The inequalities are also geographically differentiated, as those living within the municipal borders of Beirut – especially in central Beirut – have been privileged with longer prescheduled hours of electricity provided by EDL than the rest of the country.

Even though Lebanon has witnessed a ‘solar energy boom’ in the past years (Fheili and Nucho 2024; Monroe, 2023), diesel generators remain the most common way to bridge the electricity gap. The use of generator can, however, become an economic burden. Not all are able to carry it, as for the poorest segment of the society, it can take up to 88% of households’ monthly income (HRW, 2023). Those with enough wealth are able to rely on private or co-owned generators, whereas most are either connected to generators serving their apartment block or subscribe to a larger neighbourhood generator operated as a business venture (colloquially known as *ishtirak*, meaning ‘subscription’). These subscription-based generator businesses operate in a grey area. Running private generators is officially prohibited, with the EDL holding exclusive rights over electricity production, transmission, and distribution (Law No. 462/2002, see however Taha and Akel, 2024, on Law 318/2023). Yet – as is the case with other practices in Lebanon (e.g. Baumann, 2024; Saleh, 2016) – the notion of legality becomes flexible in the absence of adequate state services, compounded by a political climate marked by cronyism and clientelism. Bending regulations and laws is a common practice, often done openly – especially by those with connections to ‘the right people’. Although regulatory measures have been introduced in the generator sector, numerous operators reportedly have close ties with politicians and public officials, enabling them to act with relative impunity – and earning them the label ‘generator mafia’ (Abi Ghanem in Abu-Rish et al., 2019; Monroe, 2023; Saleh, 2023).

As the label ‘generator mafia’ underlines, there is a prevailing perception that generator operators are ‘above the law’, leaving those relying on their services at their mercy. Furthermore, the connections between the ruling class and the operators are repeatedly named as the reason why the politicians seem to hold little interest in reforming the electricity sector. Doing so would potentially undermine the mutually beneficial relationships they have with generator operators. Was the EDL to provide electricity 24/7, generators would become obsolete. The failure in introducing a reliable electricity infrastructure has thus created a lucrative business opportunity, which has only become more profitable during the past years due to the increased reliance on the generators (e.g. Cuyler, 2023). Because of these types of networks, Lebanese hold little trust in their political leaders’ ability to implement reforms or to put the ‘general good’ at the forefront of their actions. Curiously, since the end of the civil war, several plans have been drafted and a wealth of money invested in reconstructing the national electricity sector, but with few notable changes on the ground (see Verdeil, 2018). Tariq, a professional working in the electricity sector, was clear in his judgement on why the situation has not improved: ‘the main problem is the corruption’. He maintained that ‘thieves are running the country’ and according to his assessment, they have never had enough: ‘You’d think that after one million, two million they would not need more, but they keep on going’.

As an example, Tariq told of a project proposed to the government a decade ago. The plan blueprinted a micro-grid solar energy system operated through municipalities. Introducing large-scale renewable energy production would have eased Lebanon’s dependency on the fuel imported to produce electricity in the power plants, which has contributed to the economic demise in the country. For years, EDL’s budget has been in deficit and between the period of 1993 and 2020, 46% of Lebanon’s public debt was created by transfers made to cover EDL losses (Ayoub et al., 2021). According to Tariq, the group drafting the plan had prepared everything, even a loan from a foreign corporation with a reasonable payment schedule. They underlined that the investment would save the country 800 million dollars per year and thus ease the economic burden. However, in the end the government did not implement the project. ‘The same is the case with other plans for infrastructural improvements. They take the file [the project plan] and put it in a drawer’. Frustrated, he concluded that ‘you can find corruption all around the world but in other countries, they still implement the projects. Here, they take the money and do nothing’.

As has been noted (Baumann and Moore, 2023; see also Baumann and Kanafani, 2020), the short-term private solutions can ‘exacerbate the overall infrastructural and ecological predicament’ as they stall the implementation of more long-term solutions. While the state-run electricity sector is plagued by several problems – insufficient and out-dated modes of production, stealing of electricity via irregular wirings, and widespread non-payment of electricity bills – there is no question that the inability of the state to take necessary actions to reform the electricity production is behind the increased reliance on generators. Consequently, in Lebanon it is not the case that ‘the atmospheric nuisance’ is linked with the practices of the poorest (Adey, 2013); rather it stems directly from the inactions of those in power. Given EDL’s inability to ensure uninterrupted electricity provision, the majority of Lebanese have no other option than to resort to generators as an alternative source of power – contingent, however, upon their financial capacity to do so. Consequently, generators have become a major source of pollution in the country, especially in the densely built Beirut, where the toxic pollutants emitted by the constantly running generators have become inescapable feature of the urban atmosphere.

As the discussion above shows, Lebanon’s electricity crisis clearly demonstrates that failure is not simply a fact but a ‘judgement’ (Appadurai, 2016, see however Alexander, 2023), since failures can be a source of or precondition for profit. Indeed, the non-functionality of Lebanon’s electricity infrastructure has created lucrative opportunities for a wide range of actors – private enterprises offering alternative electricity solutions and for suppliers delivering diesel around the city to keep the generators running, for example. It is clear that the failures resulting from the neglect by the state treat people from different socioeconomic backgrounds unevenly, with those in the most precarious position often bearing the heaviest toll. However, what we hope to demonstrate is that when looked from an atmospheric perspective, failure becomes more encompassing. Even those profiting from the dysfunctions of the public electricity grid do *breathe* the outcomes of its failures – namely, the pollution the generators produce. As Peter Sloterdijk (2009) aptly puts it, the body ‘cannot not breathe’, rendering bodies that inhale the carcinogenic pollutants subject to atmospheric contamination. This underlines the vulnerability of the body to the effects stemming from the state’s failure to reform the electricity sector. While it is crucial to recognise the unevenly distributed precarities and intersecting inequalities related to breathing and pollution (e.g. O’Leary et al., 2023; Sharpe, 2016), the fundamental need to breathe, we argue, reveals an atmospheric dimension of infrastructural failure and state neglect, which we elaborate in the two following sections.

## The materiality of pollution

‘It’s really bad, all the pollution’, Samira remarked. We had retreated to sit beneath the shade of a tree to take a break from our chores at the NGO where she and Järvi volunteered in Spring 2024. The air felt considerably cooler under the shadow of the foliage, offering a momentary contrast to the heat radiating from the rest of the NGO’s outdoors premises, and Samira sighed, saying how much nicer it was. She continued, noting that *barra* [meaning outside but also carrying the meaning abroad] you find ‘greenery and fresh air’, whereas in Lebanon, ‘only pollution’. She mentioned a study conducted at the American University of Beirut (AUB) and noted that researchers were warning about the health effects of the bad air quality in the city. ‘Many people are getting cancer’, she went on, and named the toxic air as a reason, lamenting that even young people die ‘out of nowhere’ [without predisposing factors]. The research Samira was referring to was widely publicised in both Lebanese and international media (e.g. al-Arabiya, 2024; AJ+, 2024; al-Mayadeen, 2024; Baaklini, 2024; The Guardian, 2024; LBCI, 2024). Conducted by a research group led by chemist and MP Najat Saliba, the study politicized air pollution (da Schio and Van Heur, 2022; Kenis and Barratt, 2022; Marzecova and Husberg, 2022) by connecting it directly with the generators and with the state actors’ failure to reform the electricity sector. In their measurements, the research group found that the matter emanating from the generators had doubled since 2017, and that the amount of carcinogenic fine particulate matter, with aerodynamic diameter smaller than 2.5 μm per cubic metre, far exceeded the WHO recommendation for short-term exposure – recommendation being 15 µg/m^2^ in over a 24-hour period, and the levels in Beirut peaking at 60 µg/m^2^ (WHO, 2021, 88; Baaklini, 2024). Lebanon has also witnessed up to a 30% rise in cancer cases. While it can be too early to draw causal links between the correlating factors, oncologists have blamed the air pollution and the generators for the rise (Mehdy, 2024). Samira seemed to be drawing the same conclusion.

**Figure 1.**
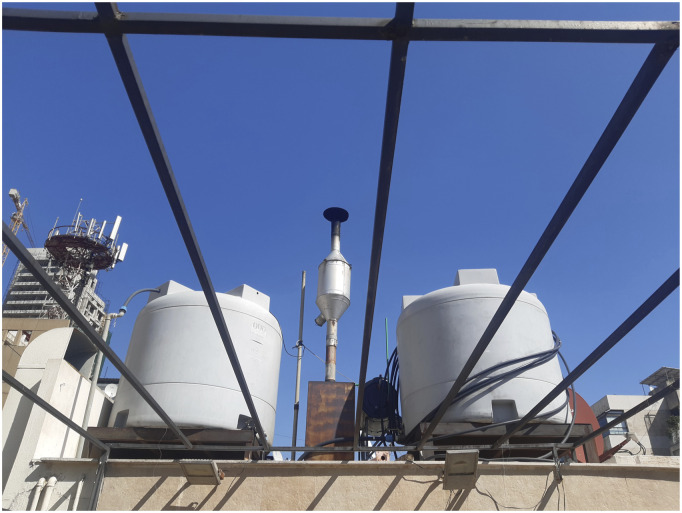
Generator on the roof of an apartment block.

Coming from the ‘outside’, I (Järvi) was able to observe and experience the change that had taken place since the economic meltdown. In 2024, at the time of the fieldwork, I continued the habit of commuting on foot. Despite its hilly relief, Beirut is a walkable-size city, and during the hours of heavy traffic it often is quicker to reach places on foot than by car. During the previous periods of work and fieldwork in the city, it was possible to evade the pollution by timing the outings to hours when the traffic was calmer, and thus when the pollution levels were lower. However, in 2024, after spending an hour strolling around the central districts of Beirut on the first day of the stay, it was obvious that something was wrong. It was Sunday and the traffic was not excessively heavy, but I could nevertheless feel my throat getting sore. It quickly became clear that that there was something in the air that caused the symptoms. The next day, when joking with a Beiruti friend Rima that breathing the pollution probably cancelled out the health benefits of walking, the conversation quickly turned to generators and pollution. Emissions, Rima continued, turned her mother’s laundry black as the neighbourhood generators kept releasing black smoke that landed on the balcony where the laundry was put to dry. This was hardly an exaggeration. In a news clip covering the increased pollution levels, a journalist demonstrated the emissions with a white handkerchief that was soiled when she wiped surfaces near a generator (This is Beirut News, 2024). Similarly, the exhaust pipes of the generator at the apartment where I (Järvi) was staying had turned black (Figure 1), and the fumes it emitted made the roof balcony – otherwise at the disposal of the residents – unpleasant to spend time in.

In face-to-face encounters, participatory research, interviews and online materials^3^, the ‘generator mafia’ and the political leaders were almost unanimously seen as responsible for the situation. A person was even joking if the leaders were ‘breathing imported air’ as the rise of cancer cases did not seem to affect them in the same manner as ‘the people’^4^. The blame was assigned through the common discourse of ‘*ween al-dawle*’ – meaning, ‘where is the state’. People in Lebanon often turn to this phrase to signal their disappointment toward the actions – or the inactions, to be precise – of the political leaders of the country. While the state is obviously present in people’s everyday lives in myriad ways (Mouawad and Bauman, 2017) – also through its indifference – for the Lebanese the phrase ‘*ween al-dawle’* is a way to express disapproval of those who would have the power to implement the needed reforms. In short, it demonstrates the frustration felt when the political leaders do not seem to care for the wellbeing of the citizens. Pollution is one of the matters considered within this frame. Lebanese curse their government for the current situation – the failure to fix the infrastructure and provide 24/7 electricity, which leaves them at the mercy of the ‘generator mafia’ that profits from EDL’s failures and pollutes the city in the process.

Consequently, although there have been attempts to regulate the generator emissions by mandating the installation of filters on exhaust pipes^5^, discussions with Beirutis reveal a widespread lack of trust. This mistrust extents to both the generator operators, who are not believed to comply with regulations that would cause them extra costs, and to the state officials, who are not believed follow-up to enforce the regulations. Consequently, when the Järvi noted – in the interview with Tariq (electricity sector professional) – that there still seemed to be many generators with no filters installed to their exhaust pipes, Tariq laughed and asked ‘how many people have you seen wearing a seatbelt in Beirut’, underlining to the general disregard of regulations. While at times encountered with humour, at times people were simply annoyed by the seeming disregard for rules and regulations. Sometimes, the annoyance was directed to other Beirutis, but often it was those in power who were at the receiving end, as they facilitated things for their friends, family and associates or left the same people off the hook, while others had to struggle with seemingly pointless bureaucracy or deal with the chaos created by the impunity granted for others. Oftentimes, the ruling class and the state as its embodiment were held directly responsible. Consequently, when chatting with Samira about the contaminated rice that had disappeared from a warehouse before officials could further inspect it (L'Orient Today, 2024), she laughed and stated ‘our country is terrible. You don’t know what you’re eating, you do not know if you’re going to be okay when you go somewhere’.

As these excerpts exemplify, the sense of unpredictability is very much ingrained in the shared understanding of what it means to live in Lebanon. On top of the daily challenges, people are constantly waiting for the next calamity to fall upon them, and one gets used to hearing speculations on how a disaster of some sort is just around the corner (e.g. Hermez, 2017). Unfortunately, there are ample precedents that validate such concerns – the most recent example being the devastating and destructive bombardment by Israel, which intensified in the final weeks of September 2024 and still claims casualties in the southern parts of the country (in October 2025). The possibility that something might go terribly wrong remains an ever-present ambience, and thus part of the everyday affective atmosphere. In comparison to extreme calamities such as the port explosion in 2020, resulting from the improperly stored ammonium nitrate that caused ‘one of the largest non-nuclear explosions in global history’ (HRW, 2022), or the aerial strikes by Israel that have devastated the country since October 2023, the threat of air pollution as the ‘silent killer’ easily fades into the background. The creeping and less spectacular violence of atmospheric pollution creates its own insidious temporality that is crucially ‘out of sync’ with the everyday matters with more immediate needs and sensibilities.

Such allochronic materiality of pollution is well exemplified in relation to 2015 mass protests on Beirut’s failing waste management. With the waste piling up on the streets also came the stench, which intensifying and expanding atmospheric volumes were harder to ignore or get used to than the less repulsive and less nauseating airborne fine particles emitted by the generators. Due to such intangible and insidious materiality – slowly violent (Nixon, 2017) and harmful through its own pace (Davies, 2019) – air pollution is easier to disregard than contaminations that have a more corporeal and spectacular presence. Oftentimes, people simply get used to its presence. While aware of the repercussions, research suggests that Beirutis tend to ignore the pollution in their day-to-day lives (Khazen et al., 2018). Consequently, albeit there are moments in which the air is politicised – for instance when people connect the pollution with ‘the generator mafia’ and the inactions of the state – the rising pollution levels have not ignited large-scale protest movements, as has happened in other contexts (Loopmans et al., 2022). Those in the city grow used to the constantly roaring generators and the smell emitted by them, and though Järvi could feel the pollution in her throat, after a while it was easy to ignore.

While at times Lebanese valorise the chaos created by the lack of rule and take pride in their ability to manoeuvre it (Hage, 2021; Monroe, 2016), the discourse of ‘*ween al-dawle*’ reveals that the state is still blamed for its absence when not acting to improve the lives of Lebanon’s denizens (see also Obeid, 2010). As many feel that they have little control over the things that transpire in Lebanon, sarcasm becomes a way to encounter the sense of incapacity to alter the situation. Samira was resorting precisely to this when she laughed at the lack of control Lebanese had in keeping themselves safe, whether it was the threat of consuming the contaminated food or breathing the toxic air in Beirut. Samira was not alone as many others constantly joked about the failing infra (sarcastically declaring ‘welcome to Lebanon’), laughed that one would not get ‘a proper Lebanese experience’ if something did not go wrong (on top of the mundane disturbances). Even the quickly altering spring weather was laughingly referred to as exhibiting ‘the typical Lebanese schizophrenia’. Importantly, expressions like this illustrate how failure becomes atmospheric not only in material sense – as a carcinogenic cloud of pollutants – but also through embodied affective atmospheres that both register and attempt to negotiate its material and inhaled presence. Here affective atmospheres do not simply reflect the passive acceptance of the need to dwell within the everydayness of failure. Rather, they serve as ways of countering failure through shared affective registers that are at once ambivalently playful and critically aware of the trouble, begrudgingly accepting the situation while simultaneously highlighting its ironic aspects. Such affective atmospheres, in short, help accept the failure by *embracing* the trouble and by *distancing* it as something one has no control over (Wylie, 2017; cf. Haraway, 2018). And precisely by doing so, the shared affections point towards the material presence of *atmospheric failure* – as pollution that one might laugh about but which one cannot avoid being immersed in through breathing.

## Breathing failure

While as a source of pollution, generators lack the mobility typical, for instance, of cars (Kenis and Loopmans, 2022), they nevertheless produce volatile atmospheres which interact with their surroundings in multiple and complex ways. Generators are just about everywhere in Beirut (see Blog Baladi, 2024), several in each street and neighbourhood. Still, the level of pollution they emit depends on the type of fuel used, the frequency of maintenance, and whether or not the owner has installed a filter. The pollution is worse in areas where business or industrial activities require electricity 24/7. In residential neighbourhoods, generators are typically turned off for several hours daily – ‘for maintenance’ but also just to save on fuel costs. There are, therefore, differences in the intensity of pollution and its effects on those within its carcinogenic spheres. Factors such as residential location, the ability to spend time outside the city (or country), and financial capacity to invest in air filtering systems or access quality healthcare and medication all contribute to unequal exposure levels and varying capacities to cope with pollution-borne diseases among Beirut’s residents.

Yet even those residing in parts of the city with comparatively better air quality, whose occupations do not require prolonged exposure to outdoor environments, and who have access to quality healthcare, cannot, as urban dwellers, fully escape the pervasive presence of polluted and moving air. Air pollution might not be democratic, as we pointed out above (see O’Leary et al., 2023), but it is of concern for everyone living in the city. The denizens of Beirut are forced to carry the consequences of inadequate electricity production, which literally is the air that they breathe. Indeed, what remains crucial in comprehending Lebanese infrastructural dysfunctions and political negligence is not simply how well the infrastructure works – whether it is able to fulfil its intended function. It is equally important to recognise, we argue, that failure works here as a material excess: as pollution with toxic particles that every person living in the city is forced to breathe and thus carry in their bodies. Consequently, embodied infrastructure means breathing the failure – its materiality, its atmosphere.

By comprehending failure in terms of material atmospheres, we join several scholars in stressing that air matters – politically, spatially, materially and ontologically (Adey, 2015; Davies, 2018; Joronen, 2025; Kenis and Loopmans, 2022; Nieuwenhuis, 2016). By flowing through our bodies, air is part of us like no other element. There is no clear boundary between the air and the breathing body, as the body is immersed in the materialities of aerial atmospheres like a ‘fish in the water’. Such pneumatological immersion renders the body dependent on the material proximities of air, with the atmosphere in turn – characterised by its fluidities, shifting concentrations, and lingering presence – continually working to materially ‘envelope’ (McCormack, 2015) and ‘weather’ (Joronen and Ghantous, 2024) its volumes. There is thus a negative immersion at play in embodying the material atmospheres: even when dwelling on contaminated soil, there is a degree of autonomy in keeping the polluted particles out of our bodies, whereas the same cannot be said of the air. The body ‘cannot not breathe’, as Sloterdijk (2009) puts it, and thus the contaminated and polluted, even weaponized air-spheres keep constantly becoming part of us through the immersive process of breathing. We could not exist without air. The air is existential, or as Luce Irigaray (1999) writes: we dwell by ‘inhabiting the air’.

Importantly – as the case of generators in Beirut well highlights – what travels through and stays in bodies is not only embodied and existential, but also political and material. While the embodied (immersion by breathing) and existential (inability to not breathe) aspects are crucial for understanding how the material failure of infrastructural politics works (as pollution) – by creating conditions that trickle down the contaminating effects through immersive atmospheres –, it is the material and political aspects that should be seen as central for thinking *pollution as a failure*. Air pollution has not only been described as the ‘silent killer’ (OHCHR, 2019); its etymology is particularly revealing as ‘pollution’ shares Latin roots with ‘corruption’ – *pollutionem* and *polluere* – meaning ‘to soil, defile, and contaminate’, as well as ‘corruption’, ‘profanation’ and ‘desecration’ (e.g. Macauley, 2005), a connection that is particularly resonant in Lebanon’s case. Infrastructural politics in Lebanon fails, not simply in providing the infrastructure in a proper way (i.e. through corruption deviating from normative expectations), but through contaminating atmospheric materialities it produces. As Marijn Nieuwenhuis (2016, 501) writes, ‘breathing bodies find themselves always materially entangled in their environment’. The infrastructural politics fails precisely in pollution – the haze of a smog that hovers above Beirut while incessantly infiltrating bodies is the *matter* of corruption and pollution.

Failure hence cannot be framed only as an affirmative condition, something that ultimately leads ‘to achievement’ (Mica et al., 2023), reveals the ‘unexpected possibilities’ (Osborne, 2019) or allows ‘the possibility of breakthrough’ (Martínez, 2019). Rather, failure must be approached by looking at how it becomes embodied, in this case through the material work it does by forcing bodies into its carcinogenic volumes of pollution. As Nucho (2019) aptly exemplifies in a paper focusing on Beirut, infrastructural ‘failure in itself does not prompt transformation’ but often shows ‘existing relations of power as sectarian patronage networks use instances of breakdown to enrich themselves or consolidate their political positions’. Promised happiness or an escape to pervasively looming ‘positivity’ (Philo, 2021), or cathartic ‘recovery’ (Dekeyser et al., 2024) might never arrive, if only one consistently ‘stays with the trouble’ (Haraway, 2018). The work the failure does cannot be reduced to empowerment, capacity, or ultimately, to recovery either. Even if people laugh about the prevalent state of failure, it shows how failure stays connected to various, even opposite affective embodiments (see Ruez and Cockyane, 2021) and atmospheres of encounter. Negativity of failure, we hence argue, stands as a material marker. It continues to circulate affects, acts, bodies, and ways of living, but also patronage networks and nomenclatures around and through itself: indifferences, judgements, laughter, frustrations, as well as normalisation *of* and distancing *from* prevalent conditions of failure. But more importantly, with every breath taken the material atmospheres of failure becomes part of those in its sphere. One does not stay with the failure, the failure keeps forcing itself upon us – it embodies us, breathes and moves with us, despite the aims to distance from its trouble.

It is crucial to recognize that, as atmospheric, political neglect keeps re-forming itself spatially and temporally. Atmospheric materiality of pollution is a moving constellation that bodies are immersed in (or breathe in) in varying ways. There are the daily rhythms of electricity outages and the different ways of using the generators (private and shared ones) that are all tied to different paces of dwelling, living and moving in the city. There are also seasonal differences that affect the need for generators as well as the local weather conditions and topographies that either intensify or reduce the smog. The ‘sticky’ materiality of the air pollution, in other words, is neither stable nor confined to the close vicinity of the exhaust pipes. While often the smog is visible from afar, and the poor air quality in Beirut is a measurable condition^6^, the pollutants become atmospheric precisely by actively enveloping the air (McCormack, 2015). Like smell that can fill the space by capturing stench volumes (Joronen and Ghantous, 2024), material atmospheres of pollution are in motion: particles move, concentrate, and linger. They engage with prevalent weather conditions in ways that eventually create what can be called smog. The smog is not a mere volumetric endgame of the infrastructural failure, a remnant that will stay put. It is an atmospheric matter in motion (Nail, 2019), moving through alleys, hills, slopes and turbulences of the weather – the wind, the rain, the warmth of the summer sun – through patterns, equilibriums and changing concentrations that recompose as much as they dissolve (see also Andrews et al., 2024). Failure, in other words, has its rhythms in localised weathering(s) of atmospheric materiality.

Consequently, the failure is at times felt less present: less thick smog, less sore throats, and less stench in the air. Infrastructural neglect, and the politics entangled with it, generate their own allochronic materialities, which divert from the rhythms of everyday, rendering pollution a disjointed matter and the politics of failure something persistently ‘out of sync’. Infrastructural failure, and the politics behind it, carry their own atmospheric temporalities and rhythms, thereby also complicating the political mobilization against the situation. This helps to further comprehend how the failure becomes a normalized daily matter. It relates not only to the need to ignore carcinogens as means to ‘get by’ or as something many feel unable to make a difference to, but precisely as something showing its presence with different intensities tied to different paces. Unlike the 2015 waste crisis, which with its lingering stench volumes and the olfactory assault on breathing bodies ignited an immediate and full-frontal protest movement ‘You Stink’, pollution and the state politics entangled with it are mediated through dispersed temporalities of moving air and shaped by various intermediaries that dilute immediacy and obscure political responsibility. The effects of changing spatial intensities and varying rhythms of aerial pollutants can often be spotted only through long-term developments of environmental contamination, degradation and health issues – through the slow violence Rob Nixon (2017) has famously talked about. This poses a challenge for grasping the scope of aerial pollutants’ effects, particularly when the situations turn from more volatile weathering(s) to more abiding micro-climates of failure. The rising incidents of cancer hint at what is to come, yet the full magnitude of toxic atmospheres currently enveloping Beirut remains to be seen. This further underlines how the atmospheric manifestation of infrastructural failure – and the politics of neglect systematically underpinning it – might constitute a challenging site for protest.

## Conclusion

In this article, we have explored the material atmosphere of failure in Beirut, focusing specifically on the one shaped by rising pollution levels. As we have argued, pollution should be seen as embodied (immersion in air by breathing) and existential (bodily inability to not breathe), but importantly also as a political and atmospheric failure. We have elaborated how in Lebanon, Beirut particularly, pollution intricately links to inadequacies of the electricity sector, which, in turn, are rooted in political negligence, wasta networks, and economic mismanagement. The reliance on the diesel generators – named as a major contributor to the alarming rise of pollution levels in the city – emerges directly out of the ongoing economic crisis that has eroded EDL’s capacity to provide electricity through the national grid. The increased reliance on generators highlights how political negligence and economic mismanagement disproportionately benefit a privileged few at the expense of the majority. This political neglect continues to circulate material atmospheres and air-borne particles, but also affects, such as indifference, frustration, and sarcasm, that emerge in relation to ways in which state neglect materializes itself in the air. Pollution thus highlights how the infrastructural politics fails, not simply in building properly functioning infrastructure, but through contaminated material atmospheres and the aerial environments they entangle bodies to.

This focus on the atmospheres of failure, we suggest, offers a way to further conceptualize how infrastructural failures and dysfunctions are experienced as embodied phenomena (Alda-Vidal et al., 2023; Nassar, 2023). Thus, it can further an understanding that moves beyond looking at the mere assembling of materiality through more-than-human dimensions. Breathing bodies, we have shown, are not merely *related* to non-human constituents (Barua, 2021), such as infrastructures (Lesutis and Kaika, 2024), but crucially *immersed* in material manifestations of political neglect. Infrastructural politics fails precisely in pollution, making the failure ‘a silent killer’ *in* the air and *of* breathing bodies. Failure, in short, becomes embodied and atmospheric: if we dwell by ‘inhabiting the air’ where the failure lingers, then failure becomes part of us through what constitutes us as bodily beings. It is through such pneumatological necessity that failure shows its most immersive and unavoidable politics.

As an atmospheric failure, pollution sticks with those dwelling in its air-spheres, making the state’s negligence of the electricity infrastructure a matter that stays with the (breathing) bodies. This intimate connection, however, does not necessarily prompt people to act on it, even when they are fully aware of the dangerous long-term effects that dwelling in the polluted city has. Lebanese do place blame on the political class that has not implemented necessary reform. They also blame the ‘generator mafia’ that has benefitted while polluting the city. Yet, beyond sarcasm and blame, a large-scale protest movement, similar to one ignited by the waste crises, is yet to emerge. As we have shown, atmospheric pollution creates more creeping and often less spectacular harm with insidious temporalities ‘out of sync’ with more immediate and mundane needs. As something tied to dispersed temporalities of moving air, pollution remains more vaguely related to political assemblages that it manifests. Indeed, state neglect, and the infrastructural failures it feeds into, are shaped by various intermediaries that can obscure political legibility and responsibility. When added to exhaustion from multiple overlapping and concurrent crises in Lebanon, failure does not necessarily motivate political change and can, as Nucho (2019) observes, readily consolidate existing power relations rather than disturb or transform them. Consequently, if other failures of the state pose more immediate sensate reactions and firm political mobilisations, fine-particle pollution is more hidden in its materiality and creeping in its temporality, which makes it easier to ignore despite the severe long-term consequences.

Conceptualizing failure as atmospheric, we hold, can also inform research on other environmental issues, such as climate change, by highlighting the geographically differentiated politicizations that emerge through various, more localized processes and atmospheric/microclimatic/weathered conditions (see Barry, 2025; Wright and Tofa, 2021). The case of Lebanon, in particular, can help in understanding how the slow violence of polluting intersects with more immediate crises and pressing matters, such as poverty caused by the economic crisis, or the existential threat posed by ongoing Israeli bombardments. In conditions marked by war and crisis, and the affective and emotional stress that comes along with them, pollution as silent killer easily remains just that, silent in the background. Thinking through the insidious and creeping material failures – from pollution and environmental contamination to nuclear waste, war chemicals and other chronic toxicants (Griffiths and Redwood, 2024; Nixon, 2017) – thus requires alternative readings of failure. That reading must move beyond the immediately pungent, repulsive and spectacular ones, even as these forms often remain entangled. Crucially, approaching failure through atmospheric pollution can help in further showing how vulnerabilities are unequally distributed – not only through corruption, crony capitalism, and poor governing (Doshi and Ranganathan, 2019; Pascucci, 2021), but also through legacies of (green) colonialism (Hughes et al., 2023; Joronen, 2025) and global flows of capital (Devine and Baca, 2020). These dynamics shape vastly uneven geographies for escaping or alleviating the consequences of living amid pollution. The focus on embodied material atmospheres, then, can offer a way to think through entanglements of crises, war, state neglect, and environmental degradation, especially the geographically differentiated and politically uneven allochronic temporalities they produce, in a more immersive and material manner.

## Data Availability

The research material utilized in this article is not stored in public data repository.*

## Notes

1. The hyper-inflation started to erode the value of Lebanese lira or Lebanese pound (LBP) in Autumn 2019, and by 2024 it has lost over 98% of its pre-crisis value. Therefore, those paid in Liras are much worse off compared to those paid in dollars, which has functioned as a parallel currency in everyday transactions since the civil war.

2. In September 2024, amid the destruction caused by intensified Israel’s aerial strikes, the fear is that the infrastructural (and economic) destruction caused by application of Dahiyeh Doctrine could push the country ‘over the edge’, that in its current economic situation, Lebanon ‘would not survive’.

3. Reddit alone has several threats on the ‘generator mafia’ and the pollution the generators produce, where people ponder online how to change the situation and threating the consequences. E.g. https://www.reddit.com/r/lebanon/comments/1ccuwie/cancer_rate_increasing_by_30_yearly_in_beirut/; https://www.reddit.com/r/lebanon/comments/hss7ai/help_us_beat_the_electricity_mafia_and_clean_the/; https://www.reddit.com/r/lebanon/comments/wgc9p1/generator_mafia_lebanon_why_its_sustained/; https://www.reddit.com/r/lebanon/comments/zcl83t/how_to_deal_with_generator_smoke_health_hazard/; https://www.reddit.com/r/lebanon/comments/1d8mhha/khayyyy_247_generator_fresh_air/.

4. ‘The strange thing is that cancer affects people, not leaders. Is it possible that they are breathing imported air?’ [Arabic]; from https://www.facebook.com/GreenpeaceAR/posts/845627310940993?ref=embed_POST.

5. Directives have been issued both on municipal and state level, the in 2022 and 2023 respectively. https://www.lbcgroup.tv/news/news-bulletin-reports/724321/generator-pollution-crisis-lebanese-environment-ministry-takes-action/en.

6. However, as an austerity measure, the Ministry of Environment closed down the nationwide national air monitoring network in 2019, and since then here has not been continuous measurements on the pollution levels (see Frakes, 2019).
